# Osteophyte Bridge Formation Correlates with Vascular Calcification and Cardiovascular Disease in Diffuse Idiopathic Skeletal Hyperostosis

**DOI:** 10.3390/jcm12165412

**Published:** 2023-08-20

**Authors:** Ryosuke Hirota, Atsushi Teramoto, Mitsunori Yoshimoto, Hiroyuki Takashima, Naomi Yasuda, Arihiko Tsukamoto, Noriyuki Iesato, Makoto Emori, Kousuke Iba, Nobuyoshi Kawaharada, Toshihiko Yamashita

**Affiliations:** 1 Department of Orthopaedic Surgery, School of Medicine, Sapporo Medical University, Sapporo 060-8556, Japan; 2 Faculty of Health Sciences, Hokkaido University, Sapporo 060-0808, Japan; 3 Department of Cardiovascular Surgery, School of Medicine, Sapporo Medical University, Sapporo 060-8556, Japan

**Keywords:** diffuse idiopathic skeletal hyperostosis, vascular calcification, cardiovascular disease

## Abstract

Diffuse idiopathic skeletal hyperostosis (DISH) is a noninflammatory spondyloarthropathy characterized by ectopic calcification of spinal cord tissue. Its etiology is possibly polygenic. However, its pathogenesis and systemic effects remain unclear. Recent studies have reported a high prevalence of DISH in heart failure patients. The authors investigated how the incidence and severity of DISH are associated with vascular calcification and the occurrence of cardiovascular events. In this retrospective chart review study, 500 patients with cardiovascular disease who underwent surgery (cardiovascular events group) and 500 patients with non-cardiovascular disease who underwent computed tomography scans (non-cardiovascular events group) were randomly selected to investigate the degree of ossification of the anterior longitudinal ligament and the incidence of DISH. We found that the incidence of DISH was higher in patients with cardiovascular events and that patients with DISH had more calcification of the coronary arteries and aorta. Next, we examined the relationship between the degree of coronary and aortic calcification, the incidence of DISH, and the degree of ossification of the anterior longitudinal ligament in the non-cardiovascular event group. The prevalence of DISH in the cardiovascular and non-cardiovascular groups was 31.4% and 16.5%, respectively (*p* = 0.007). Aortic calcification and a predominant degree of vascular calcification with a certain level of ossification of the anterior longitudinal ligament suggest some correlation between DISH and cardiovascular events. This study is important in understanding the pathophysiology and pathogenesis of DISH.

## 1. Introduction

Diffuse idiopathic skeletal hyperostosis (DISH) is a disease that occurs mainly in the spine and is characterized by calcification and ossification of the enthesis, which is the attachment site of tendons, ligaments, and joint capsules. In 1950, Forestier et al. [1] first reported senile ankylosing hyperostosis of the spine in older adults, and in 1975, Resnick et al. [2] renamed it DISH because similar changes occur in peripheral joints other than the spine. Resnick et al. [2] defined the diagnostic criteria for DISH as (A) continuous gentle calcification or ossification, described as flowing, on the anterolateral surface of four or more vertebrae, (B) relatively well-maintained disk height and absence of significant degeneration such as vacuum phenomena or osteosclerosis of the endplates, and (C) absence of bony ankylosis of the intervertebral joints, erosion of the sacroiliac joints, osteosclerosis, or bony fusion [3].

DISH occurs more often in men than women, affecting about 1.5 times the number of men than women [4]. The reported risk factors for DISH include old age, male sex, obesity, diabetes mellitus, and hyperuricemia [5,6,7]. It is known to cause ossification of ligaments, especially at the middle and lower thoracic levels [8]. Drawing an analogy to ankylosing spondylitis (AS), an inflammatory spine calcification condition, it is possible that inflammation may play a role in DISH [9]. As DISH is often found to be comorbid with metabolic disorders, it is possible that DISH is at least partly driven by a shared biochemical pathway with metabolic disorders such as hyperlipidemia and hyperglycemia [10]. The pathogenesis of DISH is likely polygenic and dependent on the interaction of multiple gene variants, epigenetics, and environmental factors. DISH is likely influenced by many polymorphisms influencing inheritance, pathology, and expression in various genes [11]. However, the pathogenic mechanism of ossification in relation to these diseases and conditions has not been clarified. The high prevalence of aortic stenosis in patients with DISH has been reported to be an independent risk factor for the occurrence of cardiovascular events, and a statistically significant association was found between DISH and aortic sclerosis based on age and sex [12]. 

The Framingham risk score is used to assess the 10-year risk of developing cardiovascular disease [13]. In a study without cardiovascular disease, patients with DISH had a significantly higher prevalence of metabolic syndrome and a significantly higher Framingham risk score when compared to the controls [14]. In a cross-sectional study, the prevalence of DISH was reported to be as high as approximately 30% in patients undergoing coronary artery bypass surgery, heart valve replacement, or those with chronic heart failure [15]. Karina et al. reported a higher rate of myocardial infarction and congestive heart failure in the DISH group than in the non-DISH group at the 10-year follow-up [16]. 

Based on these findings, we hypothesized that there is a strong relationship between the prevalence of DISH and vascular calcification and the occurrence of cardiovascular events. Recently, a continuous quantitative measure of DISH severity has been developed [8,17]. The purpose of this study was to investigate the prevalence and severity of DISH in patients with cardiovascular disease who underwent surgery and to evaluate the magnitude of vascular calcification in patients with DISH to determine the relationship between DISH, vascular calcification, and cardiovascular disease.

## 2. Materials and Methods

This retrospective chart review study involving human participants was conducted in accordance with the ethical standards of the institutional and national research committee and with the 1964 Helsinki Declaration and its later amendments or comparable ethical standards. This study was approved by the Human Investigation Committee (IRB) of Sapporo Medical University (Approval No. 322-305). Since this was a retrospective study, informed consent was not required for submission; however, the opt-out choice for this study was posted on the hospital’s website, and we did not receive any inquiries.

**Figure 1:**
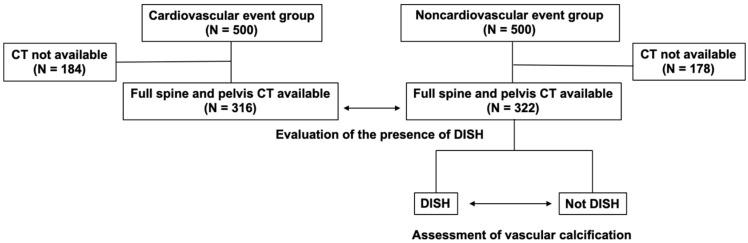
Flowchart of the study. After randomly selecting 500 patients in both the cardiovascular event group and the non-event group, we included patients who had undergone full spine and pelvic CT. We then evaluated the presence of DISH in the cardiovascular and non-cardiovascular groups. Next, we classified the non-cardiovascular group into two groups, DISH and non-DISH, and evaluated the degree of vascular calcification in each group. CT, computed tomography; DISH, diffuse idiopathic skeletal hyperostosis.

First, we randomly selected 500 patients who underwent surgery at the Department of Cardiovascular Surgery, Sapporo Medical University Hospital from 2015 to 2020 (c) and 500 patients who were transported to the hospital’s Advanced Emergency Center for reasons other than cardiovascular disease (non-cardiovascular group) (Figure 1). In each group, all patients treated during the above-mentioned period were assigned a hypothetical consecutive number, and a random number was generated by a computer, from which 500 patients were randomly selected. We included patients who had undergone full spine and pelvis computed tomography (CT). In both groups, the prevalence of DISH was diagnosed by a single spine surgeon evaluating CT images using the definition proposed by Resnick et al. [3]. We used this assessment to determine whether DISH was more common in the cardiovascular group. Using a continuous measure of DISH severity (flow score) developed by Kuperus et al., CT images were used to quantify the degree of osteophyte bridge formation across all visible vertebrae at each vertebral level of the spine [8]. From the C3/4 intervertebral level to the L4/5 intervertebral level, each level included on the CT scans was assessed segment by segment (i.e., 2 vertebral bodies with their intervertebral disc). The presence and completeness of a bone bridge could be scored in the sagittal and coronal planes as 0 = no bridge, 1 = osteophytes with ≥2 mm distance between 2 bony structures, 2 = osteophytes with <2 mm distance between 2 bony structures or connected in a maximum of 2 sagittal/coronal CT slices, or 3 = completely fused bone bridge (slice thickness 1 mm).

Each score was summed at the cervical (C3/4-C7/Th1 level), thoracic (Th1/2-Th12/L1 level), and lumbar levels (L1/2-L4/5 level) and quantified with a score for the total spine.

Next, patients in the non-cardiovascular group were assigned to one of the two groups, according to the presence or absence of DISH (Figure 1). SYNAPSE VINCENT (version 6.1 Fujifilm, Tokyo, Japan) 3D image analysis software was used to analyze the CT images for the degree of vascular calcification for all patients. Vascular calcification was assessed by using the coronary artery calcification score (CACS) [18] and the aortic calcification index (ACI) [19] by analyzing CT images. We evaluated aortic calcification by dividing the aortic arch from its origin to the aortic bifurcation into 10 equal sections and adding the ACI of the 10 axial images. Furthermore, in the non-cardiovascular group, cases were divided into five groups based on the aforementioned DISH scores, and vascular calcification was analyzed in each group. By conducting this assessment, the correlation between the presence of DISH and vascular calcification was investigated. Assessment of the presence and extent of DISH was performed by a single orthopedic surgeon, and assessment of vascular calcification was performed by a single radiologist. All data collection was done in a blinded fashion, after which the correlation between ligament ossification and vascular calcification was analyzed.

Laboratory data on admission, including glycated hemoglobin (HbA1c [%]), total cholesterol (mg/dL), and uric acid (mg/dL), were estimated. 

Statistical analysis was performed using the Student’s *t*-test and the chi-square test for comparison between the two groups, and odds ratios (OR) and 95% confidence intervals (CI) were calculated to identify the correlation between the presence of cardiovascular disease and the presence of DISH. Statistical significance was set at *p* < 0.05. All analyses were performed using the SPSS software (version 23; SPSS, Chicago, IL, USA). 

## 3. Results

### 3.1. Evaluation of DISH in the Presence or Absence of Cardiovascular Cases: Patient Background

A total of 316 patients (231 men, 85 women, mean age 70.6 years) in the cardiovascular group and 322 patients (240 men, 82 women, mean age 70.9 years) in the non-cardiovascular group had CT imaging of the whole spine and sacroiliac joints necessary to evaluate the presence of DISH. Furthermore, CT was performed to evaluate vascular calcification of the coronary arteries and aorta, respectively. 

The cardiovascular group had a significantly higher percentage of men; however, no differences were observed in mean age, height, or weight (Table 1). 

**Figure 2:**
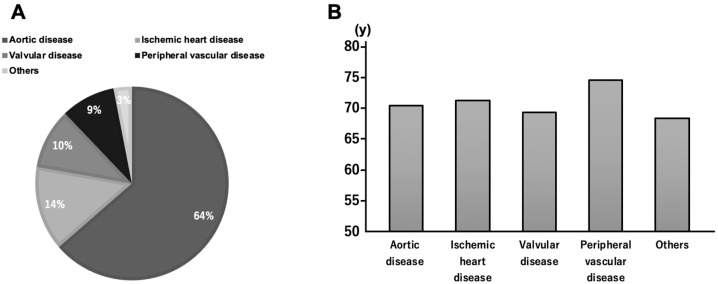
(**A**) Proportion of each disease recorded in cardiovascular surgery. (**B**) Average age of the patients with each of the cardiovascular diseases observed in our sample. The y-axis shows the average age of each disease group.

In addition, aortic diseases such as aortic dissection and aneurysms were the most common specific diseases in the cardiovascular group, followed by ischemic heart disease, valvular disease, and peripheral vascular disease. There was no significant difference in age per disease (Figure 2).

### 3.2. Percentage of DISH in the Presence or Absence of Cardiovascular Disease

**Figure 3:**
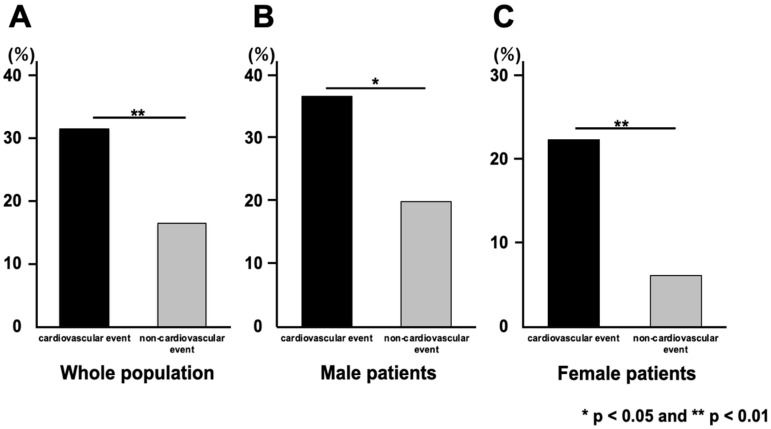
The prevalence of DISH amongst the whole study population (**A**), and amongst male (**B**) and female patients (**C**). The y-axis shows the prevalence of DISH. DISH, diffuse idiopathic skeletal hyperostosis.

A significantly higher percentage of patients in the cardiovascular group had DISH: 31.4% in the cardiovascular group and 16.5% in the non-cardiovascular group (*p* = 0.007). The prevalence of DISH among men was 36.8% in the cardiovascular group and 20.0% in the non-cardiovascular group, and the prevalence among women was 22.4% in the cardiovascular group and 6.1% in the non-cardiovascular group, with a significantly higher percentage of DISH in the cardiovascular group among both sexes (Figure 3).

### 3.3. DISH Severity in the Presence or Absence of Cardiovascular Events

Comparing DISH scores between the cardiovascular and non-cardiovascular groups, there was no significant difference between the two groups at the cervical level, but the cardiovascular group had significantly higher scores at the thoracic level (5.1 ± 1.6 vs. 2.9 ± 1.1, *p* = 0.015), lumbar level (2.1 ± 1.4 vs. 1.5 ± 0.9, *p* = 0.034), and total spine level (8.9 ± 3.3 vs. 5.8 ± 2.6, *p* = 0.017) (Table 2).

### 3.4. Association of Vascular Calcification with the Presence or Absence of DISH

Of the 322 patients in the non-cardiovascular group, 53 were in the DISH group, and 269 were in the non-DISH group. There were no significant differences in mean age, height, or weight; however, the percentage of men was significantly higher in the DISH group (Table 3).

**Figure 4:**
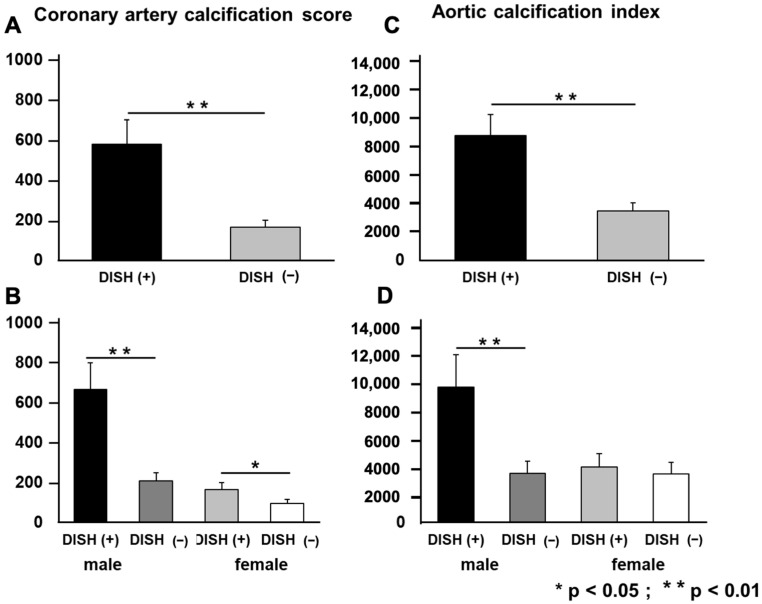
Presence of diffuse idiopathic skeletal hyperostosis and vascular calcification. The coronary artery calcification score (**A**) and aortic calcification index (**B**) for the non-cardiovascular group. The coronary artery calcification score (**C**) and aortic calcification index (**D**) calculated for each sex. DISH, diffuse idiopathic skeletal hyperostosis.

When evaluating the degree of vascular calcification, we found that the DISH group had a CACS of 580.3 ± 104.5 and an ACI of 8712.5 ± 1655.4, whereas the non-DISH group had a CACS of 166.4 ± 28.3 and an ACI of 3445.0 ± 620.1. These results were similar in both men and women, although they were particularly strong in men (Figure 4).

### 3.5. Vascular Calcification by DISH Score

**Figure 5:**
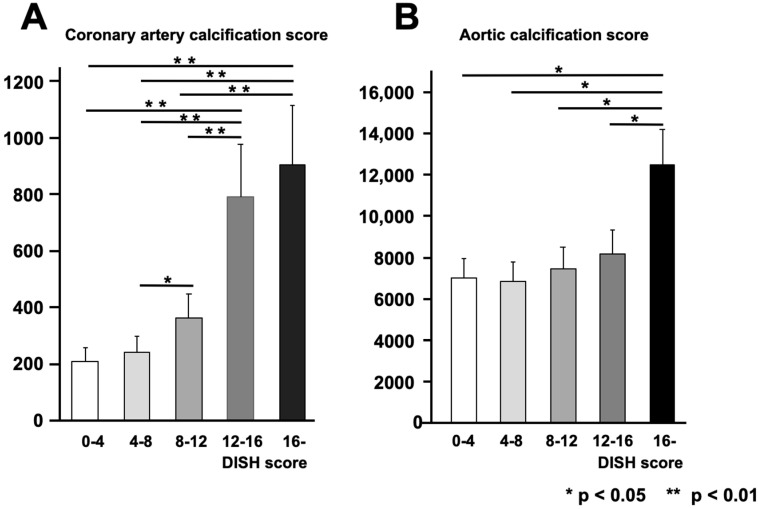
Vascular calcification evaluation by DISH score. Relationship between DISH score and the coronary artery calcification score (**A**) and DISH score and the aortic calcification index (**B**) in the non-cardiovascular group.

The DISH score was divided by four points and classified into five groups according to the severity of ossification of the anterior longitudinal ligament. In each group, coronary artery calcification and aortic calcification were evaluated (Figure 5). The higher the DISH score, the stronger the ossification of the anterior longitudinal ligament in both the coronary arteries and the aorta, especially in the coronary arteries, where the ossification of the anterior longitudinal ligament became significantly more severe when the DISH score was 12 or higher. For the aorta, ossification of the anterior longitudinal ligament became significantly more severe when the DISH score was 16 or higher.

## 4. Discussion

DISH remains a poorly characterized, diagnosed, and understood disease. Genetic and environmental risk factors for the development of the condition are not well understood, and the long-term impact of DISH on health outcomes has not been systematically analyzed. In this study, the prevalence of DISH was 31.6% (36.8% in men and 22.4% in women) in the cardiovascular group that required surgical treatment. Calcification of the coronary arteries and aorta tended to be stronger in DISH cases, indicating a significant correlation between the two conditions. A possible mechanism for the association between the two conditions is the existence of common risk factors. Diabetes, obesity, and aging are known to be risk factors for DISH as well as atherosclerosis [20,21]. Obesity and type 2 diabetes are frequently associated with hyperinsulinemia, which is also seen in patients with DISH [6]. Since insulin induces blood stem cells to differentiate into chondrocytes, hyperinsulinemia may induce cartilage formation and growth, followed by ligament ossification [5].

Patients with DISH often experience joint pain, reduced flexibility, and decreased pulmonary function [22,23], which are significantly associated with neck and shoulder pain and the use of nonsteroidal anti-inflammatory drugs (NSAIDs) [24]. Overdoses of NSAIDs and other analgesics may influence the occurrence of cardiovascular events.

In addition, growth hormone stimulates osteoblast differentiation and promotes local production of insulin-like growth factor 1, which may promote bone formation and be involved in ossification. Furthermore, it is known that adipokines such as leptin, a bioactive protein secreted from adipocytes, are associated with increased bone metabolism [22,25], and that obese rats with increased leptin receptor genes show progressive ossification of the spinal ligaments [26]. On the other hand, there is a possibility that the persistent inflammatory state that occurs in patients with DISH is itself a cause of atherosclerosis. Mader et al. [27], Weiss et al. [28], and others used echocardiography and magnetic resonance imaging to show that inflammation of the enthesis precedes the ossification process in patients with DISH. Inflammatory conditions have been shown to promote atherosclerosis [29,30,31,32], which may lead to vascular calcification and future cardiovascular events. This may be due to the complex pathway of intercellular signaling between the vascular endothelium and bone cells involving numerous mediators such as VEGF, primary fibroblast growth factor, TGF-β, and PDGF. This vascular endothelial mediation mechanism enables the targeting of osteoclast and osteoblast precursors to specific locations. These processes are also regulated by mediators and bone regulators, including cytokines, estrogen, and parathyroid hormone (PTH). Angiogenesis is an important factor in metabolic syndrome, visceral obesity, dyslipidemia, diabetes, and atherosclerosis; the association of metabolic disorders with DISH may support the role of angiogenesis [33].

Additionally, coronary artery calcification is considered a strong risk factor for cardiovascular disease. Recently, patients with DISH were found to have a significantly higher risk of coronary artery calcification, even after adjusting for age, sex, and risk factors for atherosclerosis [34]. Furthermore, it has been reported that the prevalence of DISH is higher than expected in patients with thoracic aortic aneurysms when compared to that in patients without aortic aneurysms [35]. These reports support our hypothesis that DISH itself may exacerbate the risk of cardiovascular disease.

The cervical and mid-to-lower thoracic vertebrae are the predominant sites of DISH, which results in anterolateral ossification of the anterior longitudinal ligament [1]. However, extra-spinal lesions also cause calcification or ossification of the enthesis [36]. On the trunk, bone proliferation tends to occur around the hip joints, such as the iliac crest, sciatic tuberosity, pubis, outer edge of the acetabulum, and femoral greater and lesser trochanters. In the peripheral joints, the plantar fascia, Achilles tendon attachment, and fifth metatarsal are the most common sites of calcification. The frequency of heterotopic ossification is reported to be high [37,38,39].

Recent studies have identified global excess bone formation as an important mechanism for the formation of DISH [24]. A genetic association analysis identified 10 loci associated with DISH, including several genes involved in bone remodeling [24]. Of the 10 genes, global excess bone formation is thought to be an important mechanism in the formation of DISH, GDF5 is an osteogenic protein that is associated with coronary artery disease [40], CHRDL2 is involved in diastolic blood pressure [41], and Ror2 is involved in systolic blood pressure, since many of the genetic variants associated with DISH pathogenesis are involved in circulation and metabolic dynamics such as hypertension, diabetes, and coronary artery disease, as well as bone formation [42]. This suggests that DISH and vascular calcification share genetic characteristics and the mechanisms leading to vascular calcification, and cardiovascular disease may also have contributed to DISH.

Although the majority of patients with DISH are asymptomatic, they are prone to fracture with relatively minor trauma [37], and the frequency of neurological symptoms in patients with DISH is higher than that of normal spinal trauma [37,39,43]. Early fusion surgery is recommended [44]; however, as many patients are older and have reduced reserve capacity, highly invasive surgery is risky. This is a known problem in the field of orthopedic surgery.

Our study contributes to the literature by supporting the association of DISH as an independent risk factor for cardiovascular diseases using CACS. The results of this study strongly support a genetic component of DISH, which is characterized by excessive bone formation and calcification. Based on the results of this study, the evaluation of DISH should not merely be from the perspective of a bony metabolic disease but should include a vascular evaluation with early intervention for any positive results to avoid related fatal cardiovascular events.

This study has some limitations, including the retrospective study design, DISH diagnosis performed by a single physician, potential cardiac disease in the non-cardiovascular group that could not be completely ruled out, and the fact that this is a cross-sectional study. Through this study, it is unknown if DISH preceded the calcification of coronary arteries or vice versa. The presence of similar risk factors for both diseases presents several confounding variables, which means that this study cannot provide evidence for an independent relationship between DISH and coronary artery calcification. The strengths of the study are that it is the first to confirm the interaction between DISH, vascular calcification, and the occurrence of cardiovascular events quantitatively. The reasons for the onset of DISH and its relationship with other medical diseases are still unclear, and further molecular biological studies and longitudinal clinical studies are required in the future to elucidate the mechanisms of DISH.

## 5. Conclusions

In conclusion, this study demonstrates a correlation between DISH and cardiovascular events. This is clinically significant, as it suggests that vascular evaluation and appropriate early intervention for DISH may prevent patients from experiencing fatal cardiovascular events.

## Figures and Tables

**Table 1:** Demographic characteristics of the patients included in the study.

	Cardiovascular Event Group N = 316	Non-Cardiovascular Event Group N = 322	*p*
Age	70.6 ± 14.2	70.9 ± 12.8	0.793
Sex (male %)	73.1	68.3	0.030
Height (cm)	162.1 ± 9.5	161.4 ± 8.1	0.655
Body weight (kg)	58.1 ± 12.9	60.7 ± 12.3	0.522
HbA1c (%)	6.0 ± 1.1	5.8 ± 1.0	0.381
Total cholesterol (mg/dL)	196.6 ± 21.1	198.4 ± 23.5	0.792
Uric acid (mg/dL)	5.9 ± 1.1	5.6 ± 1.3	0.263

HbA1c, glycated hemoglobin.

**Table 2:** Comparison of DISH scores by level of spine between cardiovascular and non-cardiovascular event groups.

	Cardiovascular Event Group N = 316	Non-Cardiovascular Event Group N = 322	*p*
Cervical level	1.7 ± 1.4	1.4 ± 1.3	0.367
Thoracic level	5.1 ± 1.6	2.9 ± 1.1	0.015
Lumbar level	2.1 ± 1.4	1.5 ± 0.9	0.034
Total level	8.9 ± 3.3	5.8 ± 2.6	0.017

DISH scores were significantly higher in the cardiovascular event group at the thoracic (*p* = 0.015) and lumbar (*p* = 0.034) spine levels. DISH scores for all vertebrae were also significantly higher (*p* = 0.017) in the cardiovascular event group.

**Table 3:** Demographic characteristics of the patients included in the study.

	DISH (+) N = 53	DISH (−) N = 269	*p*
Age	71.2 ± 13.5	70.1 ± 11.4	0.352
Sex (male %)	81.1	60.2	0.039
Height (cm)	163.7 ± 12.3	160.9 ± 10.7	0.517
Weight (kg)	61.8 ± 10.4	59.6 ± 9.9	0.649
HbA1c (%)	6.1 ± 1.1	5.7 ± 0.9	0.582
Total cholesterol (mg/dL)	201.5 ± 24.5	197.3 ± 22.6	0.411
Uric acid (mg/dL)	5.6 ± 1.2	5.7 ± 1.0	0.862

DISH, diffuse idiopathic skeletal hyperostosis; HbA1c, glycated hemoglobin.

## Data Availability

The datasets generated during the current study are not publicly available but are available from the corresponding author on reasonable request.
